# Antimicrobial and Antioxidant Activities of Coumarins from the Roots of *Ferulago campestris* (Apiaceae)

**DOI:** 10.3390/molecules14030939

**Published:** 2009-02-27

**Authors:** Adriana Basile, Sergio Sorbo, Vivienne Spadaro, Maurizio Bruno, Antonella Maggio, Nicoletta Faraone, Sergio Rosselli

**Affiliations:** 1 Dipartimento delle Scienze Biologiche, sezione di Biologia Vegetale, Università Federico II, via Foria 223, 80139 Napoli, Italy; E-mail: adbasile@unina.it (A.B.); 2 Centro Interdipartimentale di Servizio per la Microscopia Elettronica C.I.S.M.E., Università Federico II, via Foria 223, 80139 Napoli, Italy; E-mail: sersorbo@unina.it (S.S.); 3 Dipartimento di Scienze Botaniche, Università di Palermo, Via Archirafi, 38 - 90123 Palermo, Italy E-mail: vspadaro@unipa.it (V.S.); 4 Dipartimento di Chimica Organica, Università di Palermo, Viale delle Scienze, Parco d’Orleans II - 90128 Palermo, Italy; E-mails: antonellamaggio@unipa.it (A.M.), nic.far.ale@gmail.com (N.F.), rosselli@unipa.it (S.R.)

**Keywords:** *Ferulago campestris*, Coumarins, Pyranocoumarins, Absolute configuration, Antibacterial activity, Antioxidant activity

## Abstract

We report the isolation of several coumarins and the stereochemical assessment of some pyranocoumarins, as well as the antibacterial and antioxidant activities of the three most abundant ones (grandivittin, agasyllin and aegelinol benzoate) isolated from the roots of *Ferulago campestris* collected in Sicily and of the hydrolysis product (aegelinol). Aegelinol and agasyllin showed antibacterial activity against nine ATCC and the same clinically isolated Gram-positive and Gram-negative bacterial strains. At a concentration between 16 and 125 μg/mL both coumarins showed a significant antibacterial effect against both Gram-negative and Gram-positive bacteria. In particular the ATCC strains *Staphylococcus aureus, Salmonella thypii, Enterobacter cloacae* and *Enterobacter earogenes* (MIC = 16 and 32 μg/mL for aegelinol and agasyllin, respectively) were the most inhibited. Antibacterial activity was also found against *Helicobacter pylori*: a dose-dependent inhibition was shown between 5 and 25 μg/mL. The antioxidant activity of the coumarins was evaluated by their effects on human whole blood leukocytes (WB) and on isolated polymorphonucleate (PMN) chemiluminescence (CL), PMA-stimulated and resting.

## Introduction

*Ferulago campestris* (Besser) Grec., (*F. galbanifera* (Mill) Kock. = *Ferula ferulago* L.), *finocchiazzo*, is an annual or perennial herb with small flowers that grows in the Mediterranean area. Previous phytochemical studies on the roots of *F. campestris* collected in Egypt revealed the presence of monoterpene coumarins and sesquiterpene lactones [1]. Some *Ferulago* species have been used since ancient times in folk medicines for their sedative, tonic, digestive and aphrodisiac properties and also in the treatment of intestinal worms and haemorrhoids. Moreover, they are used against ulcers, snake bites, as well as headache and diseases of the spleen [2] and the gums obtained by incision of the roots of several species are used as spices and drugs and their use as vermifuge and for carminative disorders has been reported [3]. In continuation of our research for biologically active compounds from Sicilian medicinal plant sources [4,5], we investigated the constituents of the roots of *Ferulago campestris*. Several coumarins were isolated and the most abundant ones were tested for their antibacterial and antioxidant activities.

## Results and Discussion

**Figure 1:**
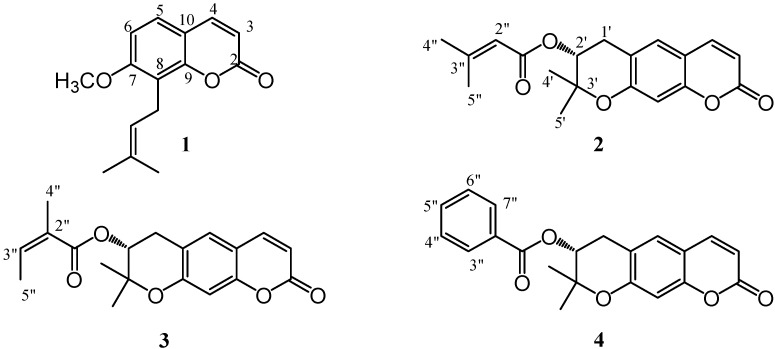
Structures of isolated compounds **1**-**4**.

Finely ground dried roots of *Ferulago campestris* (Besser) Grec. were extracted with petroleum ether and dichloromethane, and the extracts were subjected to column chromatography. From the petroleum ether extract, osthol (**1**, Figure 1), a previously known prenylated coumarin [6] was obtained. The structure of compound **1** was elucidated using 1D and 2D NMR experiments and this data, not previously reported, are given in the Experimental section.

Furthermore, three abundant coumarins **2-4** (Figure 1) differing in the ester moieties, were isolated and identified. These compounds were shown to have a pyran ring fused with the coumarin nucleus formed through a six *endo-trig* cyclization involving a prenyl chain. In fact, they showed, in their ^1^H-NMR spectra, the signals for methylene protons (H-1’a and H-1’b) and an oxygenated methine (H-2’) [7]. The difference among them was the presence of a senecioyl, angeloyl or benzoyl ester group, respectively.

In fact, in the ^1^H- and ^13^C-NMR spectra, the senecioyl group of compound **2** was identified by signals of a tri-substituted double bond (δ_H_ 5.67, s, H-2”; δ_C_ 115.60, C-2”; δ_C_ 158.39, C-3”) and two methyl groups on C-3” (δ_H_ 2.15, s, H-4”; δ_C_ 27.52, C-4”; δ_H_ 1.88, s, H-5”; δ_C_ 20.34, C-5”). Compound **3** bore an angeloyl residual, as shown by the signals of an olefinic proton at δ_H_ 6.11 (H-3”, q) (δ_C_ 127.34, C-2”; δ_C_ 139.43, C-3”) and two methyl groups (δ_H_ 1.88, s, H-4”; δ_C_ 20.53, C-4”; δ_H_ 1.84, s, H-5”; δ_C_ 15.76, C-5”). Finally, the ^1^H-NMR spectrum of compound **4** showed a benzoyl moiety (δ_H_ 7.98 *d*, H-3” and H-7”; δ_H_ 7.43 *t*, H-4” and H-6”; δ_H_ 7.56 *m*, H-5”).

These compounds were identified as the known grandivittin (**2**), agasyllin (**3**) and benzoyl aegelinol (**4**), respectively, previously reported from *Eryngium campestre* [7]. Compounds **2**-**4** are ester derivatives of aegelinol (**5**), whose absolute configuration at C-2’ was not clear. A careful bibliographical survey, indicated an ambiguous absolute configuration of C-2’. Abyshev *et al*. [8,9] reported an (*S*) C-2’ absolute configuration, although he cited the degradative determination of the absolute configuration performed by Lemmich [10]. On the other hand, Erdelmeyer *et al*. [7] indicated an (*R*) C-2’ configuration: in both cases, the reported optical rotation of the compound was negative. For further clarity, the three esters were hydrolyzed, giving the same compound with a negative optical rotation.

Consequently, we decided to determinate the absolute stereochemistry by mean of Mosher’s [11] and Horeau’s methods [12]. The Mosher esters were prepared by the standard procedure for esterification using the *S* acid chloride (*S*-MTPA-Cl) and the *R* acid chloride (*R* -MTPA-Cl). Then the ^1^H-NMR spectra were recorded and Δδ = δ(*S*)- δ(*R*) values were determined, where δ(*R*) are the proton chemical shifts on the alcoholic portion of the *R* ester (**6a**) and δ(*S*) are the proton chemical shifts of the alcoholic portion of the *S* ester (**6b**).

**Figure:**
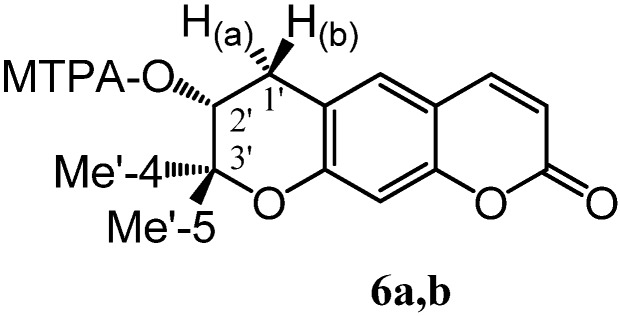


**Table:** 

Protons	δ(*S*)	δ(*R*)	∆δ
**H-1’(a)**	3.28	3.25	+0.03
**H-1’(b)**	2.99	2.87	+0.12
**Me-4’**	1.33	1.38	-0.05
**Me-5’**	1.26	1.32	-0.06

In accordance with the selection rules, we concluded that the absolute configuration of C-2’ is *R*.

**Figure 2:**
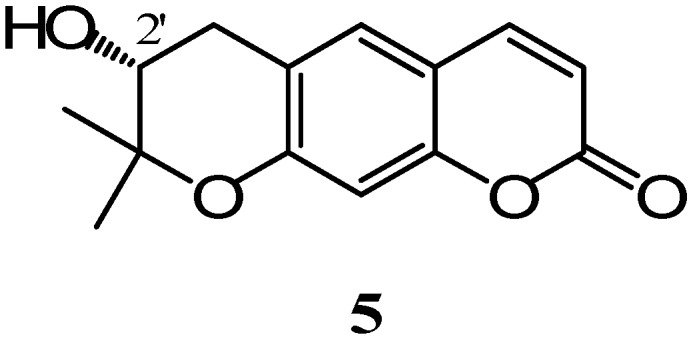
Structure of aegelinol.

**Figure 3:**
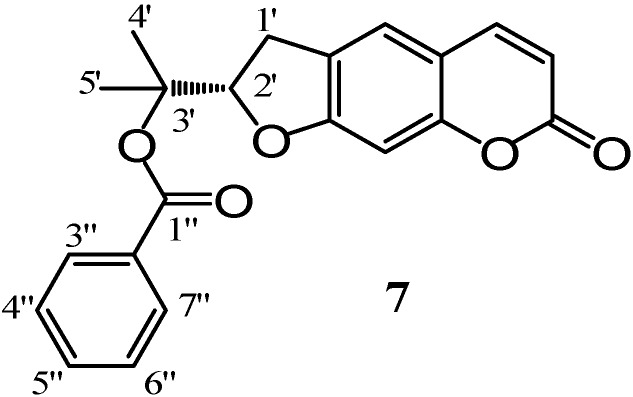
Structure of felamidin.

With regard to the Horeau’s method, compound **5** was esterified with 2-phenylbutyric anhydride (*meso*). The reaction was followed by polarimeter and α_1_**=** -1.34 (±0.029) and α_2_**= -**1.54 (±0.02) were determined. In accordance with the Horeau’s rule [(α_1_-1.1 α_2_) > 0], the absolute configuration of the C-2’ was thus confirmed to be (*R*). Therefore we concluded that structure of compound **5** is as indicated in Figure 2:

The investigation of dichloromethane extract resulted in the isolation of felamidin (**7**, Figure 3), an isomer of aegelinol benzoate, in which a prenyl chain gave a five *exo-trig* cyclization resulting in a coumarin with a tetrahydrofuran fused ring. The ^1^H-NMR spectrum showed the presence of two methylene protons at δ_H_ 3.30 (2H-1’, m) and of a benzoyl residual at C-3’. Felamidin (**7**) was previously isolated from the roots of *Ferulago isaurica* [13].

### Antibacterial activity

**Figure 4:**
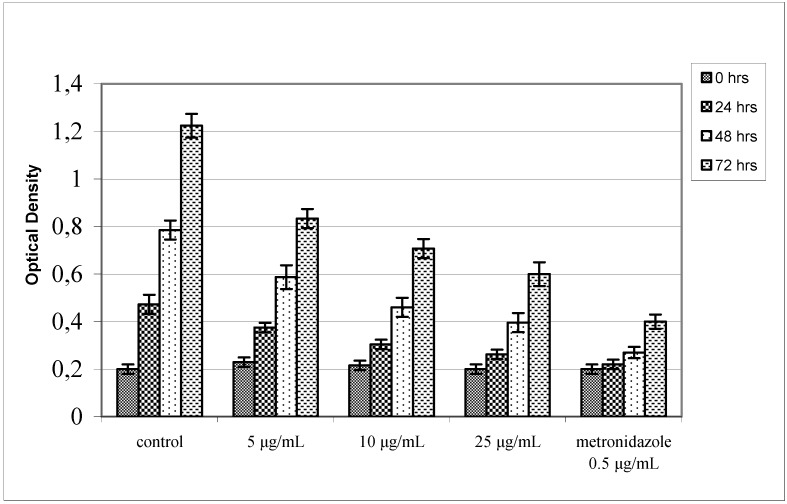
Antibacterial activity of agasyllin (**3**) against *H. pylori* growth expressed as O.D. at 450 nm. Control cultures were made culturing the bacterium without and with metronidazole.

**Figure 5:**
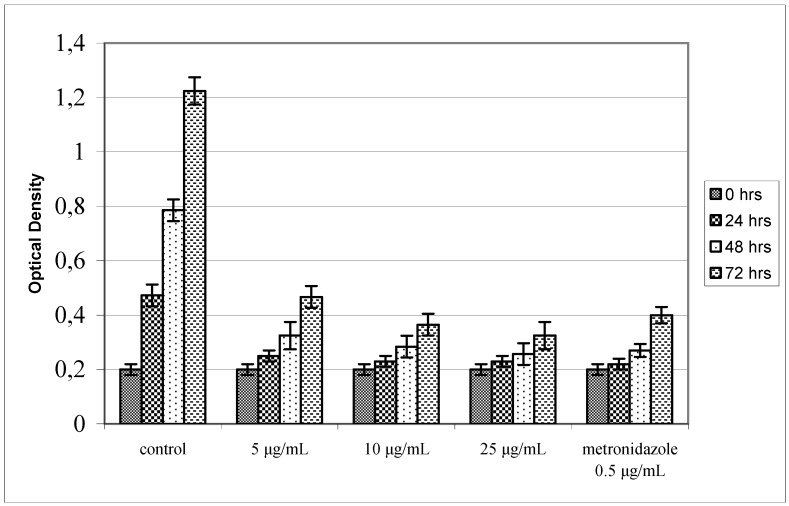
Antibacterial activity of aegelinol (**5**) against *H. pylori* growth expressed as O.D. at 450 nm. Control cultures were made culturing the bacterium without and with metronidazole.

The observed combined resistances are shown in Table 1. Among the four coumarins tested only aegelinol (**5**) and agasyllin (**3**) showed any significant antibacterial activity. At a concentration between 16 and 125 μg/mL both coumarins showed a significant antibacterial effect against both Gram-negative and Gram-positive bacteria. In particular, the ATCC strains *Staphylococcus aureus*, *Salmonella thypii*, *Enterobacter cloacae* and *Enterobacter earogenes* were the most inhibited, showing a Minimum Inhibitory Concentrations (MIC) of 16 and 32 μg/mL for aegelinol and agasyllin, respectively. The antibacterial activity of both coumarins was higher against Gram-negative than Gram-positive bacteria as reported previously from other plants [14,15]. In particular, we found a remarkable activity against *Salmonella thypii*, which is responsible for severe infections and is very often resistant to conventional antibiotics. Comparing the coumarin activities against ATCC and clinical isolated strains, we found similar effects, although the clinical isolates, except one, were resistant to more than one reference antibiotic. Antibacterial activity was also found against *Helicobacter pylori*: a dose-dependent inhibition was shown between 5 and 25 μg/mL (Figure 4 and Figure 5).

**Table 1:** Antibacterial activity (MIC values μg/mL) of compounds **2**–**5** and of reference antibiotics.

	Compounds				Antibiotics		
Organism	2	3	4	5	CTAX	PENG	TET
*S. aureus* ATCC 13709	250	32	125	16	2	0.03	2
*S. aureus* CI	250	64	250	32	R	R	R
*Ent. faecalis* ATCC 14428	125	32	64	32	R	8	2
*Ent. faecalis* CI	250	64	125	32	R	R	R
*P. vulgaris* ATCC 12454	R	64	R	32	2	4	R
*P. vulgaris* CI	R	125	R	64	32	R	R
*P. mirabilis* ATCC 7002	R	64	R	32	0.03	4	32
*P. mirabilis* CI	R	125	R	64	32	R	R
*S. typhii* ATCC 19430	R	32	R	16	0.05	4	1
*S. typhii* CI	R	32	R	32	1	2	1
*E. cloacae* ATCC 10699	125	32	64	16	R	4	R
*E. cloacae* CI	250	64	125	32	R	R	R
*E. aerogenes* ATCC 13048	125	32	64	16	R	4	R
*E. aerogenes* CI	250	64	125	32	R	R	R
*Ps. aeruginosa* ATCC 27853	250	64	125	32	16	R	32
*Ps. aeruginosa* CI	R	125	R	125	32	R	R
*K. pneumoniae* ATCC 27736	125	64	125	32	0.01	R	16
*K. pneumoniae* CI	250	125	250	64	32	R	R

CTAX = cefotaxime; PENG = Benzyl Penicillin Sodium; TET = Tetracycline; CI = Clinical Isolated; R = Resistant

This last result was of interest because *Helicobacter pylori* is a Gram-negative, microaerophilic, shaped bacterium that is free living in the mucous layer of the human stomach. This bacterium is spread worldwide, with a frequency ranging from 25 % in developed to 90 % in developing areas, but not all infected people develop illnesses. Infection from this bacterium leads to different clinical disorders including chronic gastritis, peptic ulcer and gastric adenocarcinoma [16]. *Helicobacter pylori* induced disorders depending on the inflammatory response mediated by cytokines [17].

### Chemiluminescence studies

We showed the antioxidant effects of the coumarins, grandivittin (**2**), agasyllin (**3**) and aegelinol (**5**) on the *in vitro* human PMN respiratory burst. The aegelinol benzoate (**4**) is inactive. The antioxidant activity of the coumarins was evaluated by their effects on human whole blood leukocytes (WB) and on isolated polymorphonucleate (PMN) chemiluminescence (CL) using concentration range between from 0.01 to 100 μg/mL.

Comparing the effects of coumarins on both PMN and WB chemiluminescence emission, we found that aegelinol is the most active, followed by agasyllin; grandivittin is less active, whereas aegelinol benzoate has no activity. The coumarins showed a dose-dependent and linear inhibitory activity on isolated PMN, as well as on WB CL emission PMA-stimulated and resting, showing a faster inhibition when stimulation was done. This could depend on the fact that the coumarins act like ROS scavengers and can also interfere with cellular activation mechanisms (membrane and/or cytoplasmic receptors or enzymes) [18]. The unactivated cells could be less sensitive to this down-regulation than PMA-stimulated ones. Finally, our data represent an answer to the continual demand for new antibiotics and antioxidants for the continuous emergence of antibiotic-resistant strains and the growing interest in the substitution of synthetic antioxidants with natural ones.

**Figure 6:**
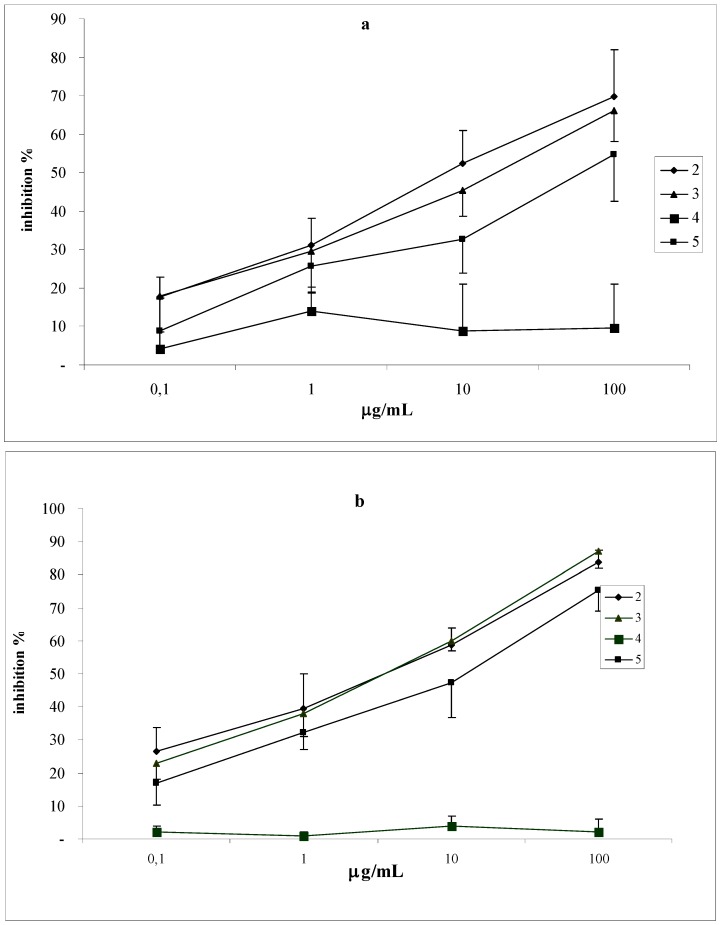
Inhibitory effects of coumarins on CL emission from resting (a) and PMA-stimulated (b) human whole blood leukocytes. Values are expressed as percent inhibition (mean ± S.D.).

**Figure 7:**
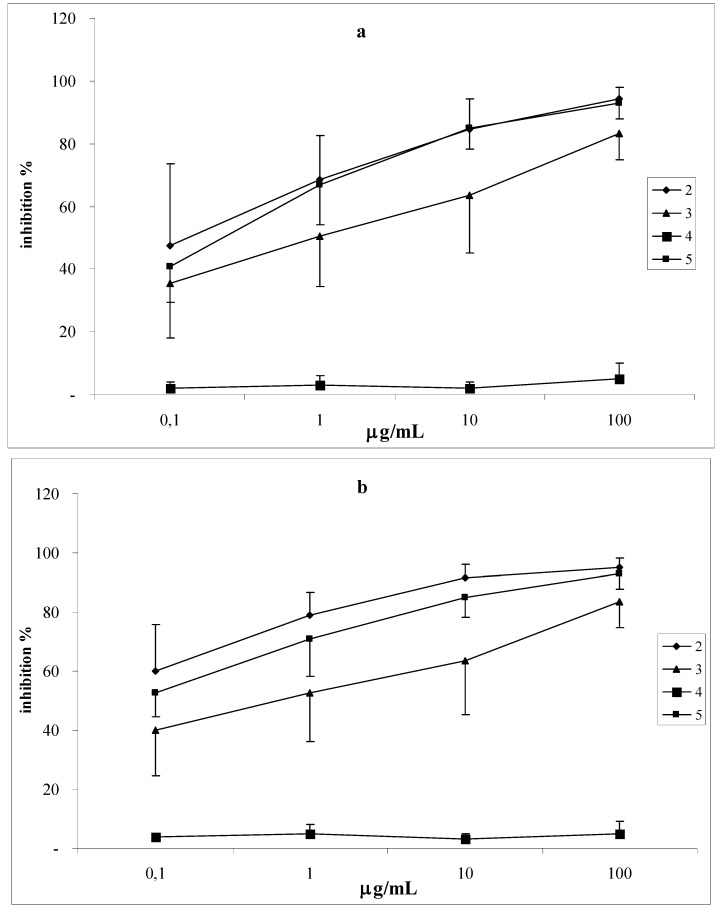
Inhibitory effects of coumarins on CL emission from resting (a) and PMA-stimulated (b) human isolated PMNs. Values are expressed as percent inhibition (mean±S.D.).

## Experimental Section

### General

NMR spectra were recorded in CDCl_3_ on a Bruker Avance series 300 MHz spectrometer. MS spectra were recorded on Shimadzu GCMS-QP2010 Plus spectrometer. Optical rotations were recorded on a Jasco P-1010 polarimeter. For open column chromatography, silica gel (Merck, 70-230) and silica flash gel (Merck, 230-400) were used.

### Plant material

Roots of *Ferulago campestris* (Besser.) Grec. (700 g) were collected at Alimena, Palermo province, Italy in July 2007 and authenticated by Professor Raimondo, Department of Botanic Sciences, University of Palermo (Italy). Voucher specimens were deposited at the Herbarium of the Botanical Gardens of Palermo (Italy) under the number PAL 07-621 (Raimondo, Schimmenti & Scafidi).

### Extraction and Isolation

The dried and chopped roots of *Ferulago campestris* (Besser.) Grec. (700 g) were extracted three times with petroleum ether (b.p. 30-60 °C) (P.Et.) and three times with dichloromethane at room temperature. The petroleum ether extract (26.5 g) was chromatographed over a silica gel column using a gradient solvent system (AcOEt-P.Et. 1:4 → 1:0). The various fractions of the initial column were subsequently rechromatographed over silica gel and silica flash columns using a gradient solvent system (AcOEt-P.Et. 1:4 → 1:0) to yield **1** (140 mg), **2** (1.2 g), **3** (1 g), and **4** (2 g). The dichloromethane extract (33.4 g) was chromatographed over silica gel column using a gradient solvent system (AcOEt-P.Et. 1:4→ 1:0) giving compounds **2** (3 g), **3** (3.6 g), **4** (5.8 g), and **7** (50 mg).

### Hydrolysis of grandivittin (**2**), agasyllin (**3**) and benzoyl aegelinol (**4**)

The esters (100 mg, about 0.3 mmol) were added to a solution of KOH in dioxane (15 mL, 3.36 g, 60 mmol, 4 M). The reaction mixture was stirred and heated to reflux for 0.5 h and was monitored by TLC (AcOEt-P.Et. 2:3). After cooling, the reaction mixture was quenched with portionwise addition of H_2_SO_4_ 10 % until acidic. The solution was extracted with dichloromethane, dried over Na_2_SO_4_ and evaporated *in vacuo*. Compound **5** was purified by crystallization (AcOEt/*n*-hexane) to obtain a white crystals (68 mg, 93 %).

*NMR data for osthol* (**1**). ^1^H-NMR (300 MHz): δ 7.61 (1H, d, *J* = 9.6 Hz, H-4), 7.29 (1H, d, *J* = 8.7 Hz, H-5), 6.84 (1H, d, *J* = 8.7 Hz, H-6), 6.24 (1H, d, *J* = 9.6 Hz, H-3), 5.22 (1H, t, *J* = 7.2 Hz, H-2’), 3.92 (3H, s, OMe-7), 3.54 (2H, d, *J* = 7.2 Hz, H-1’), 1.84 (3H, s, H-4’), 1.67 (3H, s, H-5’); ^13^C-NMR (75.3 MHz): δ 161.42 (C-2), 160.19 (C-7), 152.80 (C-10), 143.75 (C-4), 132.67 (C-3’), 126.18 (C-5), 121.08 (C-2’), 117.97 (C-8), 112.99 (C-9), 112.42 (C-3), 107.32 (C-6), 56.03 (OMe-7), 25.78 (C-5’), 21.91 (C-1’), 17.92 (C-4’).

### Spectroscopic data for compounds **2**-**5**

The physical and spectroscopic data of these compounds were in complete agreement with those reported in the literature [7].

*NMR data for compound* **6a.** ^1^H-NMR (300 MHz): δ 7.56 (1H, d, *J* = 9.3 Hz, H-4), 7.10 (1H, s, H-5), 6.75 (1H, s, H-8), 6.24 (1H, d, *J* = 9.3 Hz, H-3), 5.18 (1H, dd, *J* = 5.1, 5.7 Hz, H-2’), 3.45 (3H, s, OMe-7), 3.25 (1H, dd, *J* = 5.1, 17.1 Hz, H-1’a), 2.87 (1H, dd, *J* = 5.7, 17.1 Hz, H-1’b), 1.38 (3H, s, H-4’), 1.32 (3H, s, H-5’); ^13^C-NMR (75.3 MHz): δ 189.88 (C-1”), 161.14 (C-2), 155.95 (C-7), 154.25 (C-9), 143.01 (C-4), 128.51 (C-5), 115.04 (C-6), 113.56 (C-3), 112.95 (C-10), 104.71 (C-8), 77.23 (C-5”), 76.10 (C-3’), 73.27 (OMe-3”), 72.24 (C-2’), 55.31 (C-2”), 27.35 (C-1’), 25.26 (C-4’), 22.48 (C-5’).

*NMR data for compound* **6b.** ^1^H-NMR (300 MHz): δ 7.59 (1H, d, *J* = 9.3 Hz, H-4), 7.16 (1H, s, H-5), 6.78 (1H, s, H-8), 6.26 (1H, d, *J* = 9.3 Hz, H-3), 5.17 (1H, dd, *J* = 4.8, 6.0 Hz, H-2’), 3.43 (3H, s, OMe-7), 3.28 (1H, dd, *J* = 4.8, 17.1 Hz, H-1’a), 2.99 (1H, dd, *J* = 6.0, 17.1 Hz, H-1’b), 1.33 (3H, s, H-4’), 1.26 (3H, s, H-5’); ^13^C-NMR (75.3 MHz): δ 189.88 (C-1”), 161.10 (C-2), 156.09 (C-7), 154.34 (C-9), 142.97 (C-4), 128.61 (C-5), 114.94 (C-6), 113.65 (C-3), 113.00 (C-10), 104.85 (C-8), 77.23 (C-5”), 76.62 (C-3’), 76.21 (OMe-3”), 73.18 (C-2’), 55.35 (C-2”), 27.48 (C-1’), 25.02 (C-4’), 22.54 (C-5’).

*NMR data for compound* **6b.** ^1^H-NMR (300 MHz): δ 6.26 (1H, d, *J* = 9.3 Hz, H-3), 7.59 (1H, d, *J* = 9.3 Hz, H-4), 7.16 (1H, s, H-5), 6.78 (1H, s, H-8), 3.28 (1H, dd, *J* = 4.8, 17.1 Hz, H-1’a), 2.99 (1H, dd, *J* = 6.0, 17.1 Hz, H-1’b), 5.17 (1H, dd, *J* = 4.8, 6.0 Hz, H-2’), 1.33 (3H, s, H-4’), 1.26 (3H, s, H-5’), 3.43 (3H, s, OMe-7); ^13^C-NMR (75.3 MHz): δ 161.10 (C-2), 113.65 (C-3), 142.97 (C-4), 128.61 (C-5), 114.94 (C-6), 156.09 (C-7), 104.85 (C-8), 154.34 (C-9), 113.00 (C-10), 27.48 (C-1’), 73.18 (C-2’), 76.62 (C-3’), 25.02 (C-4’), 22.54 (C-5’), 189.88 (C-1”), 55.35 (C-2”), 76.21 (OMe-3”), 77.23 (C-5”).

### Spectroscopic data for felamidin *(7)*

The physical and spectroscopic data of this compound were in complete agreement with those reported in literature [13].

### Antimicrobial activity assays

*Microorganisms*: Nine bacterial strains from the American Type Culture Collection (ATCC; Rockville, MD, USA) were employed. They included Gram-positive (G+) bacteria: *Staphylococcus aureus* (ATCC 13709) and *Enterococcus faecalis* (ATCC 14428), and the following Gram-negative (G−) bacteria: *Proteus mirabilis* (ATCC 7002), *Proteus vulgaris* (ATCC 12454), *Pseudomonas aeruginosa* (ATCC 27853), *Salmonella typhii* (ATCC 19430), *Enterobacter aerogenes* (ATCC 13048), *Enterobacter cloacae* (ATCC 10699), and *Klebsiella pneumoniae* (ATCC 27736). The same bacterial strains, clinically isolated, were used to compare the sensitivity to the coumarins: bacteria voucher specimens are deposited at the “Cellular and Molecular Biology and Pathology Department” of Federico II University of Naples. *Helicobacter pylori* (CCUG strain) was kindly provided by the Cellular and Molecular Biology and Pathology Department of the University Federico II, Naples, Italy.

*Preparation of coumarins for antibacterial assays:* The coumarins were added with 5×10^−2^M stock solution in DMSO. They were diluted from 0.01 to 1000 μg/mL concentrations in sterile physiological tris buffer (pH 7.4, 0.05 M) [19] just before use.

*MIC determination*: The antibacterial activity was expressed as MIC (minimum inhibitory concentration) values. The MIC was defined as the lowest concentration able to inhibit any visible bacterial growth. Bacterial strains were grown on Mueller–Hinton (MH) agar plates (DIFCO, Detroit, MI) and suspended in MH broth (DIFCO). The MIC values were measured using the broth-dilution method (MH broth) [20]. Each coumarin was tested in triplicate, the experiment was performed four times.

*Anti-Helicobacter pylori activity*: *Helicobacter pylori* was cultured as elsewere reported [21], inoculated on a Brucella Agar plate containing 10% sterile defibrinated sheep blood, 5 mL Vitox (Pharmanex) and 2 mL Skirrow (DIFCO) (medium supplements) and cultured at 37 °C for 72 h. The bacterial colonies were collected and diluted to 107 colony forming units (CFU)/mL with 0.9% NaCl saline solution. The coumarins were diluted with DMSO and then added to the liquid culture medium. The culturing medium was made of 14 g Brucella broth, 1.25 mg FeCl_3_·6H_2_O, 500 mL bi-distilled H_2_O, 25 mL fetal calf serum, 5 mL Vitox and 2 mL Skirrow. Each bacterial suspension was added to the culturing medium obtaining 106 CFU/100 ml/well density. The mixture was incubated at 37 °C for 72 h [22,23]. Control cultures were made culturing the bacterium without and with metronidazole at 0.5 μg/mL.

*Chemiluminescence studies*: Luminol-dependent chemiluminescence assay represents a simple, rapid and sensitive method to identify compounds with antioxidant and anti-inflammatory activity [24].

*Preparation of coumarins for chemiluminescence assays*: The coumarin solutions were added with 5×10^−2^M stock solution in DMSO and, immediately before being used, diluted from 0.01 to 100 μg/mL in modified Krebs–Ringer phosphate medium (KRP) [24].

*Blood collection and PMN isolation*: Peripheral blood was collected from three healthy fasting donors between 08.00 and 09.00 a.m., to minimize day-time variability of phagocytic blood cell respiratory burst [25]. Samples were withdrawn by K_3_EDTA vacutainers (Becton Dickinson, Plymouth, UK). PMNs were isolated using a discontinuous gradient, consisting of 100% (density 1.1294 g/mL) and 70% (density 1.090 g/mL) isotonic Percoll (Pharmacia, Uppsala, Sweden) in calcium and magnesium-free phosphate buffered saline pH 7.4 (PBS; Sigma Chemical Co., St. Louis, MO, USA) [26].

*Luminol-dependent chemiluminescence assays*: Chemiluminescence assays were performed following the protocol described by De Sole *et al*. [24] and using an automatic luminometer (Autolumat LB 953, Berthold, Wildbad, Germany). Whole blood and isolated PMN CL emission was evaluated within 3 h after venipuncture. The reaction mixtures were prepared in 4 mL polypropylene vials. For the chemiluminescence test on resting leukocytes, each vial contained 100 μL of the various coumarin concentrations, 100 μL of diluted whole blood (WB, 1:100) or isolated PMN suspension (0.5×10^6^/mL) and sufficient KRP to yield a final volume of 1.0 mL. In activated leukocyte evaluation, samples with 100 μL of 1.5 μM phorbol myristate acetate (PMA; Sigma Chemicals Co.) were prepared in the same final volume. To exclude the possibility that the coumarins quenched the activated luminol, the following controls were included: luminol and coumarins at the various concentrations in presence or absence of PMA. The reaction temperature was 37 °C and the resulting light emission on each vial was recorded for 0.5 s, over a 90 min period. CL emission was evaluated as peaks. All measurements were performed in triplicate and expressed as percent inhibition. The effects of coumarins on phagocyte viability were determined by the trypan blue dye exclusion test [27].

### Statistical analysis

The following statistical protocol was established:

- Construction of dose–response curves:
  ○ Regression analysis with evaluation of the linearity of dose–response curves (dose logarithm as independent variable and CL percent inhibition as dependent) was drawn for each cumarin. If response regression was linear, the angular coefficient significance was calculated to verify the null hypothesis of an angular coefficient equal to zero.
- Comparison between dose–response curves:
  ○ When the curves of the coumarins were linear, the analysis of variance applied to regression (ANOVA-R) was performed to compare the angular coefficients; if one or more curves were nonlinear, analysis of variance was carried out by comparing the coumarins at each concentration.

The results of all experiments were expressed as mean ± S.D.

In all tests, values of p < 0.05 were regarded as significant.
